# Facial Impressions Are Predicted by the Structure of Group Stereotypes

**DOI:** 10.1177/09567976211024259

**Published:** 2021-11-26

**Authors:** Sally Y. Xie, Jessica K. Flake, Ryan M. Stolier, Jonathan B. Freeman, Eric Hehman

**Affiliations:** 1 Department of Psychology, McGill University; 2 Department of Psychology, Columbia University; 3 Department of Psychology, New York University; 4 Center for Neural Science, New York University

**Keywords:** face perception, individual differences, intergroup dynamics, social cognition, open data, preregistered

## Abstract

Impressions of other people’s faces (e.g., trustworthiness) have long been thought to be evoked by morphological variation (e.g., upturned mouth) in a universal, fixed manner. However, recent research suggests that these impressions vary considerably across perceivers and targets’ social-group memberships. Across 4,247 U.S. adults recruited online, we investigated whether racial and gender stereotypes may be a critical factor underlying this variability in facial impressions. In Study 1, we found that not only did facial impressions vary by targets’ gender and race, but also the structure of these impressions was associated with the structure of stereotype knowledge. Study 2 extended these findings by demonstrating that individual differences in perceivers’ own unique stereotype associations predicted the structure of their own facial impressions. Together, the findings suggest that the structure of people’s impressions of others’ faces is driven not only by the morphological variation of the face but also by learned stereotypes about social groups.

First impressions are powerfully influenced by faces. From a split-second glance at a person’s face, people readily make socially relevant inferences about that individual (Willis & Todorov, 2006), such as whether they are confident (Oh et al., 2020) or approachable (Oldmeadow et al., 2013). These snap judgments have the ability to influence critical outcomes, from election results (Hehman et al., 2014; Todorov et al., 2005) to sentencing decisions in the criminal-justice system (Blair et al., 2004; Wilson & Rule, 2015). Given the impact of first impressions, a clear theoretical understanding of how they are formed is crucial.

After decades of research, certain aspects of the impression-formation process are reasonably well understood. Modern models of face perception largely focus on morphological variation in the target’s face and propose that morphological differences elicit trait judgments along two or three fundamental dimensions of evolutionary significance (Oosterhof & Todorov, 2008; Sutherland et al., 2013). Each face, with its unique appearance, falls somewhere along those dimensions, and where a face is positioned along each dimension jointly determines the final impression that perceivers form of that face.

## Perceiver Variability in Face Impressions

Critically, the literature focusing on morphological influence on impressions has generally remained agnostic to perceiver and target identities. This is a problem because, recently, the universality of these models has been challenged on their limited generalizability to other stimuli and participant samples (Jones et al., 2021). Some research suggests that idiosyncratic experiences induce differences in the face-trait space (Stolier, Hehman, & Freeman, 2018; Sutherland et al., 2018) that perceivers use when forming impressions (Over & Cook, 2018). Namely, people who learn that two traits are associated (e.g., aggression and physical strength) should infer one trait from a face (e.g., aggression) to the extent that they infer the other trait from that face (e.g., physical strength). Because perceivers differ in these learned associations between facial features and trait concepts, the face-trait space likely varies across perceivers such that the same face elicits a different impression from one perceiver to the next.

This emerging perspective contends that top-down processes and particularly social-category knowledge fundamentally constrain how people perceive faces (Freeman et al., 2020). Different perceivers with different social identities (Kawakami et al., 2017; Sutherland et al., 2018) and stereotypical associations (Kunda & Thagard, 1996; Stolier, Hehman, Keller, et al., 2018) can evaluate the same target very differently, facilitating considerable variability in how perceivers form impressions. Consistent with these findings, recent work partitioning the variance in face impressions found that perceiver idiosyncrasies contribute a large proportion of variance across many traits (Hehman et al., 2017; Xie et al., 2019). These idiosyncrasies may reflect differences in how perceivers process, represent, and interpret features of the target’s face. Further, these differences may not be fully idiosyncratic, stemming from systematic differences in perceivers’ cognitive representations of groups.

Although the stereotyping literature has traditionally studied stereotypes in terms of semantic representations (Eckes, 2002; Fiske et al., 2002; Kunda & Thagard, 1996), the past few decades have seen a proliferation of research bridging face perception, categorization, and stereotyping. When people encounter individuals or faces from a given group, their culturally learned gender and racial stereotypes automatically activate, regardless of personal endorsement (Kawakami et al., 2017; Macrae & Bodenhausen, 2000). Yet the literature on stereotyping has generally not considered how stereotypic associations about social groups affect face-trait space specifically. Further, the literature on face impressions has generally focused on an invariant face-trait space regardless of group memberships or perceivers’ associations. Here, we took a novel approach to empirically connect these research traditions, testing to what extent perceivers’ learned stereotypes underlie individual differences in facial impressions of various group members.

**Statement of Relevance**

People are quick to form snap judgments about others, such as whether a stranger is trustworthy or competent, based on facial appearance. The prevailing view is that these first impressions are evoked by physical features of the face (e.g., upturned mouth, downturned eyebrows) in a way that is consistent for all people. However, most of this research has focused on White targets. Instead, we found that people form impressions differently depending on the target’s race and gender category—partly due to stereotype knowledge unique to each group. Our own learned stereotypes about each social group (e.g., “attractive Asian women are friendly”) individually influence the social impressions that we make of people from these groups. These results indicate that impression-formation processes are not agnostic to social identities and have implications for the differential relationships that arise between facial appearance and important outcomes (e.g., hiring, sentencing) for targets belonging to different groups.

## The Structuring Role of Stereotypes in First Impressions

Stereotypes about different social groups may give rise to distinct face-trait spaces for different groups through learned associations. Modern social-cognitive models posit that the processing of bottom-up facial features is dynamically constrained by top-down cognition, such as stereotype information (Freeman et al., 2020; Stolier, Hehman, & Freeman, 2018). Individuals have expectations about members of social categories (Fiske et al., 2002; Kawakami et al., 2017) and use this information as a template when forming impressions. For instance, Black men who appear physically larger are perceived as more threatening compared with White men of similar size, given racial stereotypes that associate Black men with aggression (Hester & Gray, 2018; Holbrook et al., 2016). Impressions of women are more homogeneous and valence laden when perceivers strongly endorse gender stereotypes (Oh et al., 2020), consistent with classic stereotyping work that finds warmth and competence judgments to be more negatively related for female than male subgroups (Eckes, 2002).

Other examples abound in recent literature: Neutral expressions on White, Black, and East Asian faces are perceived to subtly resemble different emotions (Zebrowitz et al., 2010). Facial perceptions of warmth and dominance differentially predict leadership judgments (Wilson et al., 2017) and career outcomes (Livingston & Pearce, 2009) for White and Black targets. Oldmeadow et al. (2013) found that facial cues and occupational stereotypes are integrated through shared cognitive representations of groups in a different manner across gender and age. Critically, racial stereotypes influence even basic sex categorization of faces (Johnson et al., 2012), suggesting that regardless of one’s conscious beliefs about these groups, learned associations have the potential to influence impressions (Macrae & Bodenhausen, 2000).

Together, these findings suggest that trait impressions from faces are correlated in stereotype-consistent ways across multiple social categories (Stolier, Hehman, Keller, et al., 2018). To the extent that perceivers combine stereotype information about the target with the target’s facial appearance to form impressions, we would expect the conceptual structure of different impressions to vary across social categories consistent with stereotypes. For example, if a perceiver believes “attractive” and “competent” are strongly associated for women but not for men, then that perceiver is more likely to evaluate women with attractive faces as competent relative to men.

## The Present Research

The present research is the first to formally test the similarity of the structure of group stereotypes and the structure of facial impressions that vary by group membership. In Study 1, we found that, on average, gender and racial stereotypes are associated with trait impressions inferred from other people’s faces. In Study 2, we examined the role of individual differences, finding that idiosyncratic differences in a perceiver’s stereotypes about social groups predict how that perceiver forms impressions of faces belonging to different groups. The Ryerson University Research Ethics Board approved Study 1, and the McGill University Research Ethics Board approved Study 2.

## Study 1

### Method

Study 1 tested whether stereotypes about gender and racial groups are reflected in participants’ impressions of the faces of people in those different social groups. To create the data structure necessary for this test, we collected data from two sets of participants. One set of participants formed impressions of faces belonging to six different Race × Gender groups along 14 traits (e.g., “assertive”). A separate set of participants was assessed on their stereotypical associations regarding these social groups (e.g., Black men, White women) along these same traits. We tested the overlap between impressions and stereotypes aggregated across participants.

#### Participants and procedure

##### Facial impressions

For impressions from faces, 5,040 participants from the United States and Canada completed ratings through Amazon Mechanical Turk for monetary compensation. Data were cleaned in accordance with our data-cleaning procedure on the basis of response time and frequency of repeated ratings (see https://osf.io/65tpb/). Participants were 72.6% non-Hispanic White, 10.4% Black, 5.6% East Asian, and 11.4% other ethnic minorities including people of mixed race. Because our analyses involved aggregating across perceivers, we analyzed ratings from White participants only to control for perceiver variability due to race, resulting in 290,641 ratings of trait impressions of 873 stimuli across 3,619 participants between 18 and 80 years old (mean age = 37.44 years, *SD* = 12.27; 69.2% female). To test whether conclusions were robust to this specification, we repeated all analyses while making no race-based exclusions (for a full description, see the supplementary materials on OSF at https://osf.io/6pwnm/).

These participants rated faces on 14 traits regularly used in the face-impressions literature: aggressive, assertive, attractive, caring, competent, dominant, friendly, healthy, intelligent, smart, physically strong, trustworthy, warm, and youthful (Oosterhof & Todorov, 2008; Sutherland et al., 2013). Each participant rated 60 to 90 different faces (all male or all female), of which an equal proportion were White, Black, and East Asian. Previous research indicates that these traits are used spontaneously when people form impressions. Participants rated these 14 trait impressions by responding to questions such as “How trustworthy is this person?” on Likert-type scales from 1 (*not at all*) to 7 (*very much*). Stimuli were presented in random order, and participants rated each target on only one trait so that all ratings were made between subjects.

##### Group stereotypes

For ratings of the social-group stereotypes, 360 participants were recruited from Mechanical Turk. Data were cleaned in accordance with the same procedure (https://osf.io/65tpb/): 10 participants were removed because there was no variation in their responses, and eight participants were removed for asking that we not use their data. Participants who self-reported as non-Hispanic White (73.0%) were included in analyses, resulting in a sample size of 252 participants between 18 and 80 years old (mean age = 34.91 years, *SD* = 11.28; 47.2% female).

We assessed these participants’ stereotypical associations about the social groups themselves (e.g., Asian men, Black women), absent any facial stimuli. Participants were asked to rate their associations with all crossed gender and race categories on the same 14 traits as above by responding to questions such as “Please indicate how people in society see Black men [on trustworthiness]” using Likert-type scales from 1 (*not at all*) to 7 (*very much*). Following previous research, we asked stereotypical associations in this manner to mitigate social-desirability bias (Devine & Elliot, 1995). Thus, this measure reflects participants’ learned associations about these groups and not what they personally endorse or believe. Order of group and trait presentation was randomized. Each participant rated each target group on each trait.

#### Stimuli

Our research design required a large number of stimuli. In total, stimuli consisted of 299 White (49.8% female), 295 Black (49.2% female), and 279 East Asian (46.2% female) faces. The participants who reported face impressions rated real facial stimuli from a variety of standardized databases, including the Chicago Face Database (Ma et al., 2015) and the Face Research Lab London Set (DeBruine & Jones, 2017). (For the full list, see the supplementary materials at https://osf.io/6pwnm/.) All stimuli depicted frontal views of faces with neutral expressions. Faces were resized to 611 pixels wide by 430 pixels high and presented against a plain background.

#### Analytic approach

Our goal was to examine the relationship between group stereotypes and impressions of individual faces. We used *representational-similarity analysis*, an approach previously used to compare inferential relationships between trait adjectives and social impressions (Lay & Jackson, 1969) and that has recently been applied to impressions of faces (Stolier, Hehman, Keller, et al., 2018). This approach conceptualizes the face-trait space as a matrix of weighted relationships between traits (e.g., correlations) that are commonly spontaneously inferred from faces (Stolier, Hehman, & Freeman, 2018; Stolier, Hehman, Keller, et al., 2018) and does not combine traits to form a set of static factors. Therefore, independently of how traits correlate differently across social groups, we tested whether correlations among face impressions were related to correlations among stereotypes within each social group.

In a supplementary analysis, we confirmed an assumption of our statistical approach, which was that the face space varied across different Race × Gender groups. Accordingly, we fitted three-, two-, and one-factor models consistent with previous research (Oosterhof & Todorov, 2008; Sutherland et al., 2013) for all race and gender groups using a confirmatory factor analysis in a structural equation framework. Results indicated poor fit and differential fit across race and gender groups, respectively, supporting our assumption that these models were noninvariant by target group. Results from an exploratory parallel analysis were consistent with this result, revealing that a different number of factors underlie the impressions of different race and gender groups (i.e., they were not equivalent). Full descriptions and results of these analyses are available at https://osf.io/6pwnm/.

##### Restructuring face ratings

**Fig. 1.**
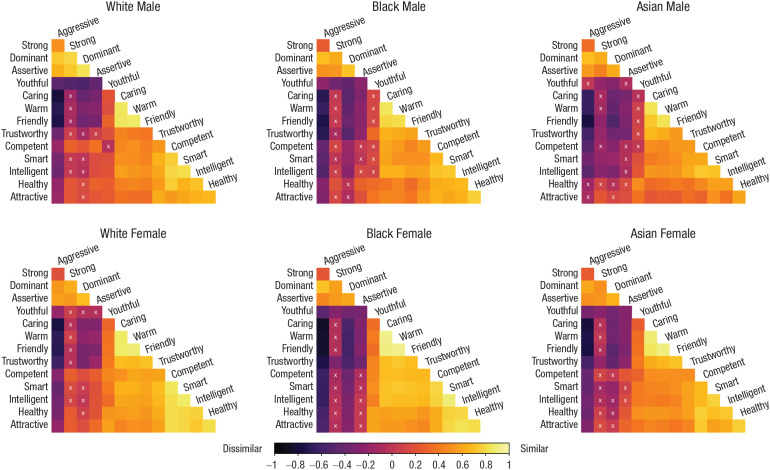
Pearson correlation matrices for White participants’ impressions of 14 traits related to male and female White, Black, and East Asian faces (Study 1). “X” denotes a nonsignificant relationship (α = .05). Matrices are sorted using the hierarchical clustering order based on the White male matrix.

Because different groups exhibited different numbers of factors and patterns of traits mapped to factors, it was appropriate to adopt our model-free approach to comparing race and gender groups, allowing for comparisons at the trait level. To this end, we followed the procedure from Stolier, Hehman, Keller, et al. (2018) to restructure the data for this analysis. We created a 14 × 14 trait-correlation matrix for each of the six groups, producing separate correlation matrices for ratings of female and male White, Black, and East Asian faces (Fig. 1). We removed repeated trait-pair correlations from the upper diagonal of each matrix. Values were Fisher’s-*z* transformed to allow for comparison across social groups. Each matrix was converted to a single column vector with 91 rows of trait-pair correlations in which each row represented a single trait-pair relationship (e.g., strong–aggressive) within a single social group (e.g., the correlation between ratings of “strong” and “aggressive” when viewing Black male faces). These 91 × 1 vectors for each social group were then combined into a single 546 × 1 vector representing all the correlations from ratings of faces. Data are available at https://osf.io/dytxs/.

##### Restructuring group-stereotype ratings

**Fig. 2.**
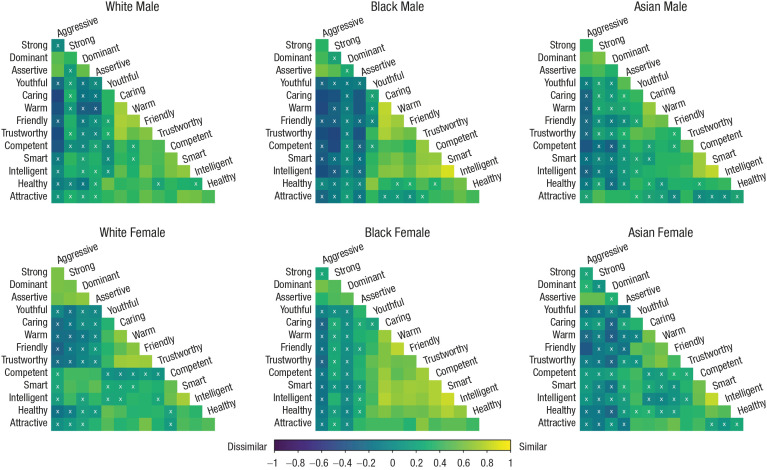
Pearson correlation matrices for White participants’ abstract impressions of 14 traits related to female and male Whites, Blacks, and East Asians (Study 1). “X” denotes a nonsignificant relationship (α = .05). Matrices are sorted using the hierarchical clustering order based on the White male matrix.

We restructured the group-stereotype data from the second group of participants in an identical manner. The 14 × 14 Pearson correlation matrices of stereotypical trait ratings of each social category in the abstract (Fig. 2) were converted to 91 × 1 single-column vectors and then combined into a 546 × 1 vector. Correlations were Fisher’s-*z* transformed for comparison. Again, each matrix contains intercorrelations between pairs of trait ratings. For example, for White male targets, the correlation between “warm” and “caring” represents the average association between participants’ ratings of White men (as a group) on warmth and caring. Thus, these matrices represent stereotypical representations of the social categories aggregated across perceivers.

### Results

#### Similarity of face-trait and group-trait spaces across groups

Our primary goal was to compare the spaces of face impressions with group stereotypes. A positive relationship between the face impressions and the group impressions would indicate that the group-level stereotypes are associated with impressions of faces. The face-trait ratings and the group-trait ratings were combined into a 546 × 2 matrix to examine this relationship. Because we were now correlating correlation matrices, Spearman’s ρ was used instead of Pearson’s *r* to evaluate the Fisher’s-*z-*transformed correlations (Kriegeskorte et al., 2008; Stolier, Hehman, & Freeman, 2018). Critically, to ensure that mean relationships between traits were not driving effects (e.g., dominance and physical strength have a more positive correlation than dominance and friendliness across all social categories), we subtracted out the average correlation of each trait pair across all six social categories. Thus, the final Spearman coefficient captured the extent to which group-level stereotypic associations uniquely relate to shifts in the face-trait space.

**Fig. 3.**
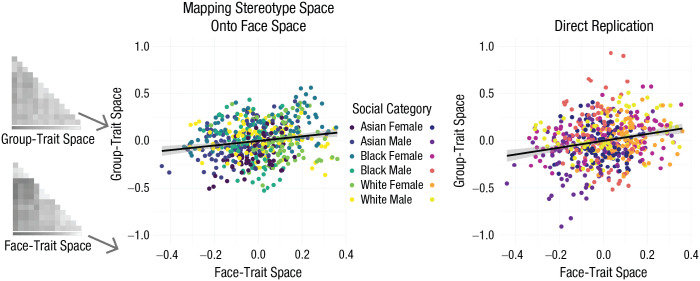
Comparison of the face-trait space with the stereotypical group-trait spaces (from a separate sample) after subtracting out the average correlation between Trait A and Trait B across all six social categories (Study 1). Results are shown separately for the main analysis and the direct replication. The slope depicts the extent to which differences in group-trait spaces uniquely overlap with differences in face-trait spaces. Error bands represent standard errors. The matrices at the left illustrate how trait-pair correlations from face ratings and stereotype ratings map onto the *x*-and *y*-axes, respectively.

Supporting our hypothesis that group stereotypes shape the impression-formation space, trait-pair correlations from ratings of faces were positively correlated with trait-pair correlations of abstract ratings of groups, ρ = .164, *p* < .001, 95% confidence interval (CI) = [.082, .245], suggesting that the trait space of stereotypic associations for a particular group (e.g., to what extent Black men as a social category are rated similarly on trustworthiness and dominance) is significantly similar to the “space” of our facial impressions of people from that group (e.g., to what extent Black male faces are rated similarly on trustworthiness and dominance; see Fig. 3).

##### Direct replication

We conducted a preregistered direct replication (https://osf.io/vzb48)^1^ to increase our confidence in the relationship. Data collection and cleaning procedures were identical to those in the previous analysis: We recruited 304 additional participants who evaluated social categories in abstract terms, removing 20 participants for having no variation in their responses and seven participants for indicating that we should not use their data. Analyses included participants who self-reported as non-Hispanic White (71.2%), resulting in a sample size of 195 participants between 19 and 70 years old (mean age = 39.69 years, *SD* = 12.47, 53.1% female). Data are available at https://osf.io/dytxs/.

Replicating our previous analysis, results indicated that trait-pair correlations from ratings of faces were positively correlated with trait-pair correlations of group ratings in the abstract, ρ = .204, *p* < .001, 95% CI = [.125, .285], a stronger relationship within the confidence interval of the previous estimate (see Fig. 3).

Together, our results reveal that stereotypical associations of traits across social categories are linked with how people form impressions of those targets. For example, to the extent that “youthful” and “competent” are more strongly positively associated for Asian women than White men, Asian women with youthful faces are more likely to be perceived as competent (likewise, competent Asian women are more likely to be perceived as youthful) relative to White men.

##### Robustness check

Previously, we restricted analyses to White participants. To test whether conclusions were robust to this specification, we repeated all analyses while making no race-based exclusions. This resulted in a total of 4,984 participants rating faces, 344 participants rating groups in the abstract, and 274 participants rating groups in the replication data set. Results were nearly identical, suggesting that the associations between group stereotypes and the impression-formation space are robust across perceiver ethnicity. (For a full description, see https://osf.io/6pwnm/.)

#### Supplementary analysis

Another assumption in the above analyses was that there is variation within the trait-pair correlations across social groups in the first place. In other words, this correlation might emerge from the same relative relationship between face impressions and group-level stereotypes if trait-pair correlations for both were equivalent across all social groups. To conclude that different stereotypes about different social groups give rise to different face impressions, it is important to confirm that variation in the face-trait space exists. Although the initial confirmatory and exploratory factor analyses (reported in the supplementary materials at https://osf.io/6pwnm/) essentially revealed that this is indeed the case, we sought to confirm meaningful variation within the same statistical framework used previously.

To this end, using the face-impressions data only, we restructured the 546 × 1 face-impression data to a 91 × 6 matrix in which each column was a single social group. The rows continued to represent trait-pair correlations from faces. We then compared these spaces using a repeated measures analysis of variance (ANOVA) in a 2 (target gender: female, male) × 3 (target race: White, Black, East Asian) design. This allowed us to examine whether the face-trait space—comprised of correlations between various trait pairs (e.g., warm–competent, warm–attractive)—differed significantly as a function of targets’ race and gender.

Mauchly’s test, χ^2^(2, *N* = 182) = 10.52, *p* = .005, indicated a violation of sphericity for race. This is a test of statistical assumptions but in this case directly informed our hypothesis because the rejection of the null hypothesis for Mauchly’s test indicated that the variance of the differences in trait-pair correlations was not homogenous across racial groups. In other words, certain groups had significantly less variance in their face-trait space: Some groups’ facial impressions were more strongly interrelated (i.e., more homogeneous) than other groups’. For the race factor, we report Greenhouse-Geisser corrections below.

Consistent with our confirmatory and exploratory analyses (reported at https://osf.io/6pwnm/), results from the 2 (target gender) × 3 (target race) repeated measures ANOVA for face ratings indicated that intercorrelations between trait pairs were not equal across race and gender. There was a significant main effect of target gender, *F*(1, 90) = 19.12, *p* < .001, η_*p*_^2^ = .18, and target race, *F*(1.80, 161.94) = 26.27, *p* < .001, η_*p*_^2^ = .23, qualified by a marginally significant Gender × Race interaction, *F*(2, 180) = 2.59, *p* = .078, η_*p*_^2^ = .03 (Table 1, left).

**Table 1.** Estimated Marginal Means From a Repeated Measures Analysis of Variance on the Six Trait-Pair Correlation Matrices for Face Ratings and Abstract Ratings of Groups in a 2 (Target Gender) × 3 (Target Race) Design (Study 1)

Variable	Homogeneity of face-trait space			Homogeneity of group-stereotype space		
	*M*	*SE*	95% CI	*M*	*SE*	95% CI
Gender						
Female	.305	.061	[.185, .425]	.300	.029	[.243, .357]
Male	.234	.051	[.132, .336]	.265	.033	[.198, .331]
Race						
Asian	.223	.052	[.121, .326]	.225	.026	[.173, .277]
Black	.257	.062	[.134, .381]	.328	.041	[.247, .409]
White	.328	.054	[.220, .436]	.294	.030	[.234, .354]
Gender × Race						
Asian female	.263	.055	[.153, .373]	.190	.030	[.130, .250]
Black female	.277	.072	[.135, .420]	.419	.038	[.343, .495]
White female	.375	.056	[.263, .486]	.291	.031	[.229, .354]
Asian male	.183	.049	[.085, .281]	.260	.029	[.202, .318]
Black male	.237	.054	[.130, .344]	.237	.047	[.143, .330]
White male	.282	.054	[.174, .390]	.297	.034	[.229, .365]

Note: CI = confidence interval.

Results indicated that, on average, the associations between different pairs of trait ratings (e.g., competent–attractive) inferred from faces differed across targets’ race and gender. Because the unit of analysis was the correlation of trait pairs, and results were the averages of these correlations, results can be interpreted as overall homogeneity of the trait space for each group. For instance, because the average trait-pair correlation is higher for women (mean *r* = .305) than for men (mean *r* = .234), we can interpret this as evidence that all traits are, on average, more interrelated for women than for men.

We performed the same restructuring and analysis for the group-stereotype data. Mauchly’s test, χ^2^(2, *N* = 182) = 8.59, *p* = .014, indicated violations of sphericity for race (similar to the previous analysis) but also for the Gender × Race interaction, χ^2^(2, *N* = 91) = 7.30, *p* = .026. Thus, we can infer unequal variances in trait-pair correlations across Race × Gender groups. Applying Greenhouse-Geisser corrections, we found that results of the 2 (target gender) × 3 (target race) repeated measures ANOVA for trait ratings of social categories indicated that intercorrelations between trait pairs were not equal across race and gender, similar to the previous analysis for trait ratings of faces. There was a significant main effect of target gender on the correlations of trait pairs, *F*(1, 90) = 4.98, *p* = .028, η_*p*_^2^ = .05, and a significant main effect of target race, *F*(1.83, 164.83) = 9.72, *p* < .001, η_*p*_^2^ = .10, qualified by a significant Gender × Race interaction, *F*(1.85, 166.86) = 26.09, *p* < .001, η_*p*_^2^ = .23 (Table 1, right).

Both of these results support our underlying assumption that the face-trait space and the group-trait space are not equivalent across social groups and lend credence to our interpretation of the relationship between the face-trait space and group-trait space. Thus, targets of different social categories evoke distinct stereotype associations, which are consistent with shifts in the trait space for facial impressions of those targets.

## Study 2

Study 1 examined face-trait and group-trait impressions aggregated across perceivers, and therefore the association reflects consensual stereotypes and impressions regarding race–gender groups. However, individuals differ in their stereotype knowledge and endorsement. Study 2 more stringently tested our hypothesis by examining this association within subjects. Specifically, we examined whether perceivers’ idiosyncratic stereotypical trait associations for each group predicted their face-trait spaces (i.e., correlations among trait impressions inferred from faces) for targets belonging to those groups.

### Method

#### Participants

We recruited 400 participants from Amazon Mechanical Turk. Data cleaning following our previous procedure (https://osf.io/65tpb/) resulted in a final sample of 181 participants between 18 and 73 years old (mean age = 38.56 years, *SD* = 11.87; 58% male). Because the analysis was within subjects, we included all individuals regardless of race and/or ethnicity, resulting in 114 non-Hispanic White, 18 non-Hispanic Black, six non-Hispanic East Asian, 22 Hispanic White, 11 Hispanic Black, and 10 selected aboriginal/indigenous, Pacific Islander, South Asian, Biracial, or other-race participants.

#### Procedure

Participants rated faces in a 2 (gender: female, male) × 3 (race: White, Black, East Asian) × 6 (trait: aggressive, attractive, friendly, healthy, intelligent, physically strong) mixed methods design with repeated measures on both the race and trait factors. We collected a reduced number of traits because of concerns about participant fatigue in the within-subjects design.

Participants first rated White, Black, and East Asian faces that were either male or female on all six traits by responding to questions such as “How attractive is this person?” on Likert-type scales from 1 (*not at all*) to 7 (*very much*). Unlike in Study 1, participants rated each target on multiple traits. Traits were presented in blocks, and order of trait presentation was randomized across participants. Facial stimuli were presented at random within each trait block (and reshuffled across trait blocks) to minimize the effects of serial dependence.

In the second part of the task, participants reported their stereotypical trait associations for each social category. Participants were asked to indicate how they thought the “average person in North America” would believe any given pair of traits was linked for each social category, expressed as a likelihood that a person with one trait would have another trait. Following previous research (Stolier et al., 2020), we asked participants to respond to questions such as “How likely is an aggressive Asian man to be attractive?” on Likert-type scales from 1 (*not at all*) to 7 (*very much*). The order of trait presentation as well as their internal ordering within the prompt (e.g., whether “aggressive” or “attractive” appeared first in the sentence) were randomized by trial and participant.

#### Stimuli

Study 1 required a large number of target stimuli, and diverse databases with minor variations in photograph standardization were included. To test generalizability and that any effects were artifacts of these different databases, we had participants in Study 2 rate color frontal photographs of faces with neutral expressions from only the Chicago Face Database (Ma et al., 2015). Each participant rated 30 unique photos of one gender—10 from each racial group. To maximize generalizability given the more limited sample, we randomly sampled stimuli from a larger pool of 120 photos (40 per racial group) on a by-participant basis. Across both female and male targets, and across all participants, a total of 240 stimuli were used. Faces were resized to 611 pixels wide by 430 pixels high and presented against a plain background.

#### Analytic approach

Because of the within-subjects nature of our design, we analyzed data in a multilevel framework. To compare the stereotype-trait space with the face-trait space within perceivers, we restructured the face-rating data using a procedure similar as in Study 1, with the additional step of nesting ratings within participants. For each participant, we created a 6 × 6 trait-correlation matrix for the three groups (female or male; White, Black, and East Asian). We estimated the trait-pair correlations (e.g., friendly–attractive) for each group (aggregating across all stimuli targets of each group) and then Fisher’s-*z* transformed them to allow for statistical comparison. These trait-pair correlations from face ratings were then joined with the stereotypical trait associations for each group from the second part of the task.

This procedure resulted in a data set in which each row contained the target’s social category (e.g., White female), a trait-pair correlation from ratings of faces (e.g., friendly–attractive), and a rating of the stereotypical pairwise association of those traits for White women (i.e., the perceiver’s rated likelihood that a White woman with one of those traits would have the other trait, expressed on a Likert-type scale from 1 to 7). This final variable was group-centered within perceivers.

##### Preliminary analysis

An assumption prompting Study 2 was that individuals would vary in their trait-pair associations. To test this directly, we built a cross-classified null model in which trait-pair correlations were nested within both participants and trait pairs. This approach partitions the variance between and within the clusters of the model and allowed us to calculate an intraclass correlation coefficient (ICC; Raudenbush & Bryk, 2002) representing the proportion of variance attributable to a portion of the model (i.e., between perceivers, between trait pairs, or within perceiver and trait pair). For example, perceiver ICC is calculated as the proportion of variance attributable to between-perceiver differences. Using this approach, we determined how much variance in the trait-pair correlations from face ratings between any given pair of traits was attributable to perceiver differences as opposed to the trait pairs themselves (i.e., the extent to which correlations between certain trait pairs varied more than others).

Results produced a perceiver ICC of .04 and a trait-pair ICC of .26, indicating that 4% of the variance in the correlation of trait pairs (from face ratings) was coming from between-perceiver differences, whereas 26% of this variance was coming from differences among the trait pairs in our study (e.g., friendly–attractive, strong–intelligent).

This preanalysis was important because it indicated that across perceivers, the correlation of trait impressions inferred from faces did not vary much (4%). Within each perceiver, this correlation may still vary as a function of each perceiver’s stereotypical trait associations for each group. Thus, for the main analysis, we centered the stereotype-association variable within each perceiver’s mean to focus on within-perceiver variation. Furthermore, the large trait-pair ICC indicated that this cluster would need to be included in the main analysis to account for heterogeneity in the correlations across different trait pairs.

Repeating this process, we calculated ICCs for the trait-pair correlations from stereotypes. Results indicated that 11% of the variance in stereotypical trait associations was attributable to the perceiver, 19% of this variance was attributable to the specific trait pairs involved, and 15% of the variance was attributable to the interaction. This preanalysis therefore provided support for including perceivers as a cluster in our primary analysis.

##### Relationship between face-trait space and group-trait space

Testing our primary hypothesis, we examined whether stereotypical trait associations idiosyncratically predicted the face-trait space for each perceiver. Given six trait ratings per target, this amounts to 15 unique trait pairs by 181 participants by three target racial groups, resulting in 8,145 observations nested in 181 participants and 15 trait pairs. Perceivers’ stereotypical trait associations (i.e., rating of the likelihood that a target who possesses a particular trait would also have another trait) were mean centered within each perceiver and included as a Level 1 predictor in the model:

$$\begin{array}{l} \mathrm{Level}\;1:Y_{ijk}=\beta_{0jk}+\beta_{1jk}\\ \;\;\;\;\;\;\;\;\;\;\;\left({\mathrm{StereotypeTrait}}_{ijk}-{\bar{\mathrm{StereotypeTrait}}}_{cwc}\right)+R_{ijk} \end{array}$$

$$\mathrm{Level}\;2:\beta_{0jk}=\gamma_{000}+\gamma_{010}{\bar{\mathrm{StereotypeTrait}}}_{j}+U_{0j0}+U_{00k}$$

$$\beta_{1jk}=\gamma_{100}+U_{1j0}$$

At Level 1 of the model, $Y_{ijk}$ is a correlation between face ratings on a pair of traits by perceiver *j* on trait-pair *k* (e.g., attractive–intelligent), now conditional on that perceiver’s stereotypical trait association of those traits (per Race × Gender group). The intercept, $\beta_{0jk}$ , is the expected value of this correlation across all targets at the average level of each perceiver’s stereotypical pairwise trait association (e.g., “How likely is an attractive White woman to be intelligent?”) across all groups. $\beta_{1jk}$ represents the correspondence between a perceiver’s stereotypical pairwise trait associations unique to each group and the correlation of their face ratings of targets from that group (e.g., attractive–intelligent for White women). Because perceivers’ stereotypical associations are mean centered within each perceiver (i.e., centered within cluster [CWC]), values on this variable represent the unique variation in each perceiver’s stereotype associations across different Race × Gender groups.

At Level 2, each perceiver’s intercept, $\beta_{0jk}$ , is an outcome modeled as the grand-mean pairwise correlation for all faces, $\gamma_{000}$ ; the between-perceiver effect of stereotypical trait associations, $\gamma_{010}$ ; each perceiver’s residual from the grand mean across all trait pairs, $U_{0j0}$ ; and the residual of each trait pair from the grand mean across all perceivers, $U_{00k}$ . $\beta_{1jk}$ models the similarity between stereotype-trait space and face-trait space within perceivers. $\gamma_{100}$ is the average increase in the pairwise correlation of face ratings with every 1-unit increase in the stereotypical pairwise association of those traits within each perceiver. The residual, $U_{1j0}$ , represents the variation of perceiver *j* around this average slope.

We hypothesized that perceivers’ stereotype-trait associations for each group predicted how they formed impressions from faces. Thus, we expected the fixed effect, $\gamma_{100}$ , to be significant. On the basis of the preliminary analyses and the results of Study 1, we expected this relationship to hold for targets of all social groups. This relationship may be stronger for some groups than others for which we had no directional hypotheses.

Finally, to estimate the variance in the face-trait space explained by stereotypical trait associations, we used the general *R*^2^ formula developed by Rights and Sterba (2019) for use in multilevel models. Because there are currently no extensions of the framework to cross-classified data structures, we adopted the formula for noncluster-mean-centered models (see Table 5 and Appendix A2 of Rights & Sterba, 2019) and modified the matrices to reflect the cross-classified data structure. For the R code, see https://osf.io/dytxs/.

### Results

**Fig. 4.**
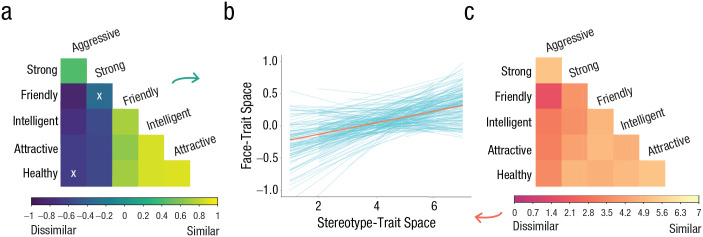
Comparison of stereotype- and face-trait spaces (Study 2). For each perceiver evaluating each group, a correlation matrix of face-trait impressions (a) and group-trait associations (c) was constructed. We tested the within-subjects relationship between these values (b). Blue lines signify the slope for each perceiver, and the orange line signifies the grand average across perceivers. Although the analysis was carried out using Fisher’s-*z*-transformed correlations from face ratings in a multilevel model, the untransformed correlations are depicted for illustrative purposes. In (a), “X” denotes a nonsignificant relationship (α = .05).

Replicating Study 1 in a within-subjects framework, results indicated that perceivers’ stereotype-trait space predicted significant differences in the face-trait space ( $\gamma_{100}$ = .040, 95% CI = [.027, .047], β = 0.104, *p <* .001; see Fig. 4). Furthermore, perceivers’ idiosyncratic stereotype content unique to each group explained 3.8% of the variance in structural relations within the face-trait space, whereas 24.4% of the variance was explained by other between-perceiver differences as well as differences in the correlations across trait pairs.

Though we had no directional hypotheses, we additionally tested whether the relationship between the stereotype-trait space and the face-trait space was consistent across all Race × Gender groups. Results were consistent across all groups, with the exception of Asian women. For full reporting, see https://osf.io/6pwnm/.

One methodological point critical to the interpretation of these results is that the stereotypical trait-pair associations were centered within perceivers and clustered within different trait pairs. Thus, regardless of the trait being evaluated, within-perceiver variation in stereotypical trait associations is still associated with how perceivers form impressions from faces. The contribution of idiosyncratic stereotypes to variance explained in this sample is small. Because participants reported stereotype knowledge instead of personal endorsement of stereotypes, this may be a conservative estimate of the link between stereotypes and the trait space generally. Nevertheless, it is an important theoretical proof of concept that when individuals evaluate individuals from different social categories, knowledge of group stereotypes influence their impressions.

## General Discussion

We present the first direct evidence suggesting that group stereotypes constrain the structure of trait impressions inferred from faces. The impression-formation spaces of different Race × Gender groups are not the same, varying in ways consistent with stereotype associations distinct to each group. Study 1 demonstrates this pattern across perceivers, revealing how culturally consensual stereotypes are linked to the average impression-formation space of each group. Study 2 demonstrates the same phenomenon within perceivers, showing that perceivers’ idiosyncratic stereotype associations predict variation in their face-trait space when evaluating targets from different groups. For example, to the extent that a perceiver believes “attractive” and “intelligent” to be more strongly associated in White than in East Asian women, that perceiver may be more likely to evaluate White women with attractive faces as intelligent relative to East Asian women. Critically, this work contributes novel evidence that the structure of facial impressions overlaps with stereotypical associations, independently of the specific traits being evaluated. We empirically connected the literatures on face impressions and stereotyping, finding that the structure of trait evaluations conforms to perceivers’ stereotypical trait associations of the target’s group.

It is important to stress that these data are cross-sectional, which limits causal inference. Yet drawing from theory, we speculate that the group-stereotype space constrains social impressions drawn from faces. Our results converge with those of recent studies finding that the face-trait space varies across group boundaries, such as gender (Oh et al., 2020; Sutherland et al., 2015), nationality (Jones et al., 2021; Sutherland et al., 2018), age (Oldmeadow et al., 2013), and race (Wilson et al., 2017), and supports the emerging perspective that individuals’ lay beliefs about personality shape the structure of the face-trait space during impression formation (Freeman et al., 2020; Over & Cook, 2018; Stolier, Hehman, Keller, et al., 2018). Under this framework, trait impressions from faces can be understood as mappings between morphological features in “face space” and conceptual relations in mental “trait space” arising from learned experiences.

One limitation is that our assessment of stereotypes and face impressions could in theory be nonindependent if participants imagine a face when they provide abstract ratings of groups. However, we believe this is unlikely because participants rated what the “average person” believes about any group, potentially encouraging more belief-based semantic representations rather than one’s own mental imagery of exemplars.

Given a lack of diagnostic information about a target’s attributes (e.g., competence), an initial stereotypic expectation based on group membership may shape other trait inferences according to stereotypical associations in the perceiver’s trait space (Nisbett & Wilson, 1977). Computational models are helpful for understanding impression formation because they illustrate that mappings in face space (e.g., symmetry, skin coloration) and trait space (e.g., attractive, trustworthy) dynamically influence one another (Freeman et al., 2020), which converges with the well-documented finding that facial impressions are both highly intercorrelated (Nisbett & Wilson, 1977; Oosterhof & Todorov, 2008) and highly variable across perceivers (Hehman et al., 2017; Hönekopp, 2006). Critically, accurate perception is not required for these associations to be influential because perceivers observe, recall, and integrate information into existing schemas in a selective and biased manner (Kawakami et al., 2017).

Social impressions from faces have important real-world implications within the political (Todorov et al., 2005) and legal (Blair et al., 2004; Wilson & Rule, 2015) systems. We found that when people are forming an impression, trait inferences are differently correlated across race and gender in stereotype-consistent ways. Thus, to the extent that physical strength and trustworthiness are negatively associated for Black men but unrelated for White men, sentencing decisions, which are influenced by how trustworthy a target appears, are more likely to be influenced by other attributes (e.g., physical strength) for Black than White male defendants. Given that defendants with faces stereotyped to be crime congruent are more likely to be found guilty (Macrae & Shepherd, 1989), idiosyncratic stereotypes in impression formation may contribute to systematic discrepancies in conviction rates across groups.

Furthermore, although the present research demonstrates variability in the face-trait space among the social categories represented here, the theoretical implications extend beyond these groups. The impression-formation space may vary by evaluative context, mood, situational affordances, and stereotypes about other groups. Given that the associations between trait words vary even on a perceiver-by-perceiver basis (Stolier, Hehman, Keller, et al., 2018), the utility of dimensional models of social perception that aggregate across perceivers or targets may be limited. Currently, the literature lacks a topography of how other factors systematically shift the space of social impressions.

Finally, although the present research indicates that current models of facial impressions are not fully generalizable, future research is needed to understand why the trait space shifts across racial and gender groups. Although we present evidence that stereotypes are associated with these shifts, the small percentage of variance explained by stereotypes alone indicates that other factors are likely important. Future research can integrate these other sources of variance to better understand group differences in impression formation.

## Conclusion

In summary, the present work synthesizes and advances the impression-formation and intergroup literatures by examining the extent to which group stereotypes constrain first impressions from faces. We demonstrated that the impression-formation space varies across female and male White, Black, and East Asian categories, partly because of stereotypic associations with these groups. These findings inform our understanding of how and why perceivers form impressions of diverse targets differently on the basis of social identity. Perceiver stereotypes uniquely predict the impression-formation space for each group, suggesting that group differences arise early in the person-perception process. These results have implications for the differential relationships that arise between facial appearance and important outcomes (e.g., hiring, sentencing) for individuals belonging to different groups.

## Transparency

*Action Editor:* Eddie Harmon-Jones

*Editor:* Patricia J. Bauer

*Author Contributions*

All the authors conceived the research and designed the methodology. Data were curated by S. Y. Xie and E. Hehman, and data were analyzed by S. Y. Xie, J. K. Flake, and E. Hehman. S. Y. Xie wrote the first draft of the manuscript. All the authors reviewed and edited the manuscript, and all the authors approved the final version for submission.

**Open Practices:** All data and analysis code have been made publicly available via OSF and can be accessed at https://osf.io/dytxs/. The design and analysis plans for the direct replication performed in Study 1 were preregistered at https://osf.io/vzb48 (see Note 1 for explanation of deviation from the preregistration). This article has received the badges for Open Data and Preregistration. More information about the Open Practices badges can be found at http://www.psychologicalscience.org/publications/badges.

## Notes

1. The effect-size *r* to be reproduced was erroneously reported as .818 in the preregistration. In the original study, we conducted an additional step of subtracting out the average correlation between traits across all six race–gender categories, which produced a Spearman’s ρ of .164. Thus, ρ = .164 is the effect size that we intended to replicate. This does not change the results of the replication. If we omit this additional step of subtracting out the mean correlation across race–gender groups and simply compare *r* = .818 with the (unsubtracted) correlation obtained in the replication, the effect is still replicated.
